# Electricity in Medicine and Surgery

**Published:** 1883-02

**Authors:** 


**Electricity in Medicine and Surgery.** By GEO. C. PITZER, Professor of the Theory and Practice of Medicine in the American Medical College of St. Louis, etc.. etc. St. Louis, 1883.

The author states in his preface that the object of this work is to furnish the medical student with a book containing the principal practical facts embraced by the subject of electricity and electro-therapeutics. He has certainly succeeded in his endeavor, and the medical man who is not much accustomed to the use of the battery will find this a most useful work, superior to many of the larger works, because everything is made as plain and simple as possible so that the novice in the use of electricity in the treatment of disease will find here just the information he needs.

